# The medicolegal, psycho-criminological, and epidemiological reality of intimate partner and non-intimate partner femicide in North-West Italy: looking backwards to see forwards

**DOI:** 10.1007/s00414-019-02061-w

**Published:** 2019-04-23

**Authors:** Georgia Zara, Franco Freilone, Sara Veggi, Eleonora Biondi, Dario Ceccarelli, Sarah Gino

**Affiliations:** 1 Department of Psychology, University of Turin, Via Po 14, 10123 Turin, Italy; 2 Present Address: Institute of Criminology, Sidgwick Site, University of Cambridge , Cambridge, CB3 9DA UK; 3 Laboratory of Criminalistic Sciences “Carlo Torre”, Department of Public Health and Pediatrics, University of Turin, Corso Galileo Galilei, 22, 10126 Turin, Italy; 4 Present Address: Department of Health Sciences, University of Piemonte Orientale, via Solaroli 17, 28100 Novara, Italy

**Keywords:** Femicide, Intimate partner violence, Non-intimate partner violence, Risk factors, Contentiousness, Overkilling

## Abstract

This paper addresses femicide in Italy. The assumption is that femicide is not a discrete act of killing a woman. It is assumed that depending on the types of relationship between the victim and the perpetrator (e.g., known versus unknown, intimate versus acquaintance), the risk processes may differ. When femicide involves the killing of an intimate partner, it is likely to be characterized by sustained and escalating intimate partner violence (IPV) that can reach its climax with extreme acts of violence that lead to intimate partner femicide (IPF). Eighty-six cases of femicide that occurred in North-West Italy between 1993 and 2013 were examined in this study. Findings suggest that femicide was disproportionately perpetrated by intimate partners (current or past), rather than strangers. IPF was likely to be the epilogue of an abusive relationship, with high levels of contentiousness and conflicts being the frequent significant precursors. Non-intimate partner femicide (NPF) was more likely to be characterized by antisocial or predatory motives, highly frequent when the victims were prostitutes.

These preliminary findings suggest that joint scientific, professional, and political efforts are paramount in order to address strategies aimed at assessing the differential risk of IPV early in time so as to prevent it from escalating into IPF or NPF and to provide the appropriate support for victims and their families.

## Introduction

Despite all the prevention campaigns, violence against women remains a major public health problem worldwide [1, 2], with a global prevalence of 30% [3, 4] and with a proportion of 38.6% of women killed by their intimate partner and a proportion of 6.3% of men killed by their intimate partner [5, 6]. Intimate partner violence (IPV) is defined as any form of actual, attempted, or threatened physical and psychological abuse perpetrated by a man or a woman against someone with whom he or she has, or has had, an intimate and affective relationship [7, 8]. Non-intimate partner violence (NPV) is the actual or threatened violence perpetrated by a stranger or by a person with whom the victim has only a superficial relationship. Both forms of violence include gross violation of a person’s integrity and their right to autonomy and security that often anticipates the extreme act of killing [9]. The rates of recidivism for IPV are estimated to be high [1] and to range between 15% and 60% across studies [10–14]. Other studies have advocated that the risk of IPV recidivism is also higher when controlling for the antisociality of the perpetrator [10, 15], for their psychopathic traits [16], and for their psychological terrorism [17].

Empirical and clinical studies [18–20] have always shown that antisocial and violent past behavior is by far the most robust predictor of future violent behavior, which can have terrible consequences leading to secondary victimization [21, 22]. It was demonstrated that antisocial perpetrators were more than twice as likely to recidivate in IPV compared with the family-only perpetrators and that antisocial perpetrators were more likely to recidivate in both physically and non-physically violent IPV, to recidivate within the first year after their reported index crime, and to recidivate faster than family-only perpetrators in non-physical IPV [12].

A 2014 survey of 42,000 women across 28 EU member states [23] showed that the lifetime prevalence of IPV was on average 22%. A WHO multi-country study on women’s health and domestic violence shows estimated peaks of prevalence of IPV of up to 71.0% in countries such as Ethiopia [24].

Given this scenario, scientists and scholars have thought that there is the need for coordinated work from multiple agencies to address any form of violence against women and to combat it effectively and directly before it escalates into femicide^1^ [13].

The term “femicide,” first coined by Corry in 1801 to signify the killing of a woman [14], was retrieved, two centuries after, to symbolize a gender-based murder [25, 26] and to specifically convey the murder of a woman by a man for reason of hate, disdain, passion, or sense of ownership over women.

A systematic review by Stöckl and colleagues [27] on the prevalence of intimate partner homicide at a global level suggested that for the 66 countries for which data were obtained, an overall 13.5% of violent deaths were caused by an intimate partner; this proportion was six times higher for femicide (38.6%) than for homicide (6.8%). Other studies have tried to examine the emotional and existential costs of IPV that often anticipate and represent a prelude to femicide. It is not unusual that prior domestic violence and/or IPV are by far the most significant risk factors for femicide and familicide [28].

If the aim is to tackle IPV at its bud, then attention should be devoted to the risk processes that foster a proviolence attitude [29], the exploitation of women [30], and interpersonal violence between heterosexual intimate partners [31] and between same sex couples [32]. In some instances, femicide is followed by familicide [33] and by the suicide of the perpetrator [34].

Despite violence not always leading to the death of women, the consequences of violent acts could be dramatic and debilitating to say the least [35]. Physical and psychological long-term effects of violence against women involve public health concerns [36], which have substantial human and economic costs [37].

The major caveats are over the diverse criteria for assessing the risk [38–40], and some uncertainties concern the heterogeneity of perpetrators [12, 41] and victims [42–44] and the risk factors [45, 46] that sustain this type of violence across time, geographical areas, social classes, and cultural levels. Interdisciplinary efforts [47] and interprofessional collaboration [48, 49] seem to be required to address the complexity of IPV and of femicide [35] and their causes and consequences.^2^

Despite global awareness being raised, more laws coming into force, and much investment being made to prevent femicide, the extent of the problem does not seem to have waned, and violence within intimate relationships seems to continue to be present in different and in more insidious forms [51]. This is the case in countries like Italy.^3^ Many women are still killed by men in Italy. In numerous cases, these were men with whom the victims were in a relationship; in many situations, the relationship was abusive and the women were humiliated and had been deprived of any dignity and autonomy for years. Very little is known about how and why many women stayed in an abusive relationship that then became a lethal trap.

The Italian scenario of femicide was investigated in two studies conducted respectively by Bonanni and colleagues [52] and Moreschi and colleagues [53]. In the first study, the Italian situation was compared with the international one, and four cases of femicide were specifically chosen and analyzed in order to profile a specific subgroup of femicide. In all murders, victims and perpetrators were bound by a relationship, and the victim’s decision to break off the relationship represented the trigger that fostered the killing rage of her partner. In the second study, cases of femicide in North-East Italy were examined, and researchers explored the circumstances and risk factors surrounding femicide, the type of weapons used, and any experience of threats and violence, prior to the femicide.

The aim of this paper is to examine cases of femicide in North-West Italy, specifically in Turin and its metropolitan area of about 1,000,000 inhabitants, which occurred between 1993 and 2013 and which involved only males as perpetrators and females as victims. No cases of same-gender IPV were present in the sample.

Primarily, the goal is to explore femicide and the risk processes involved by also differentiating intimate partner femicide (IPF) from non-intimate partner femicide (NPF). Secondly, the interest is to compare femicide in North-West Italy with findings highlighted by Moreschi and colleagues [53] in North-East Italy during the same temporal span of 21 years.

For the purpose of the current study, IPF is defined as the killing of a woman by a person (usually her husband or ex-partner) with whom she had been emotionally close [54]. IPF is likely to be the dramatic epilogue of a relationship that escalated to an unsustainable condition of contentiousness [55], hostility, and extreme violence [35]. NPF is defined as femicide by strangers or acquaintances.

## Materials and methods

In order to meet all the ethical standards, the researchers followed all possible procedures to ensure confidentiality and fair treatment of data and information and to guarantee, at each stage of the research, that the material was treated with respect and discretion. The research protocol was organized according to *The Italian Data Protection Authority Act* n. 9/2016, art. 1 and 2 (application and scientific research purposes) and art. 4 (cases of impossibility to inform the participants, e.g., deceased people), to *The Code of Ethics of the World Medical Association* (Declaration of Helsinki) for experiments involving humans (2013), and to the recent General Data Protection Regulation (GDPR) (2018), and it was carried out in line with the Italian and the EU code of human research ethics and conduct in psychology, forensic pathology, and legal medicine.

The research was approved by the Bioethics Committee of the University of Turin (protocol n. 191414/2018).

The data were collected both at the Institute of Legal Medicine, which signed a letter of intent with the Department of Psychology (University of Turin) in 2016 to support this research, and at the Archive of the Morgue of Turin whose Director authorized data collection.

All data were anonymized and made unidentifiable; data were also numerically coded for statistical purposes. The software package IBM SPSS Statistics version 25 was used.

Through a retrospective review of database of records from the Institute of Legal Medicine and the Archive of the Morgue in Turin, this study identified all cases of femicide in Turin and in the metropolitan area, between 1993 and 2013. We excluded cases of women’s natural death or suicide. The first phase of the study consisted of identifying the victims of femicide, while in a second phase, information about age, profession, previous involvements in violence or criminality, dynamics of the aggression, and types of relationships between perpetrators and victims were gathered. Information on injuries and victims’ bodies, included in the written reports of coroners, was collected at the Legal Medicine Institute and at the Morgue of Turin. Forensic files from the Court of Turin were also examined, along with files from the archive of the medical experts involved in the cases.

All information collected was classified according to the following dimensions: temporal trends and locations of the femicide, victims’ and perpetrators’ profession and qualification, victims’ and perpetrators’ age and nationality, medicolegal aspects on the murder (e.g., weapons used, location of deadly injuries on the body, extension of the damage caused, and presence of defense wounds), motives for killing, and perpetrator’s modus operandi and reactions after killings. Types, intensity, and duration of the relationship between the victim and the perpetrator provided researchers with information to differentiate between IPF and NPF. Contentiousness between the victim and the perpetrator and overkilling were also investigated. These dimensions are relevant in the assessment of (1) the type of emotional involvement between the victim and the perpetrator over time and the presence of violent incidents previously reported to the police and/or of frequent rows or conflicts (*contentiousness*) and of (2) the dynamics of femicide by examining the extent of force or action used to cause death (*overkilling*).

This is the first study in Italy that attempts to assess contentiousness and overkilling in IPF and NPF. Contentiousness implies the presence of negative, intense, and enduring emotional strain between people in a relationship (leading to intolerance, coercion, control, and dissatisfaction) [35, 55]. Overkilling is described as the excessive use of force that goes further than what is necessary to kill. If the victim sustained multiple injuries that went beyond those necessary to cause her death, this was counted as overkilling [35]. This description has been converted into scientific analysis criteria according to Jordan and colleagues [56], who suggested that overkilling involves multiple injuries resulting in one or more causes of death (i.e., multiple gunshot wounds) or multiple wounds distributed over two or more regions of the body [57]. Two independent raters carried out the categorization of data into motives of crime, i.e., risk factors for IPF and NPF, and the assessment of contentiousness and overkilling. Separate variables were created to indicate the presence (coded as 1) or absence (coded as 0) of contentiousness and overkilling in each femicide case as described in the scientific and clinical literature. According to the literature on IPV typology [58], perpetrators are considered a heterogeneous group of individuals [59], as are their victims [60]. A typology^4^ provides useful and practical information to identify different etiological mechanisms of partner and non-partner violence [62] and differential risk factors [63].

Motives for killing included six categories such as passion crime (e.g., the inability to accept the breaking up of the relationship and jealousy), multi-problematic relationships (e.g., continual acrimony, psychological terrorism, and contentiousness), familial problems (e.g., health problems, financial preoccupation, and job loss), mental disorders, predatory crime, and general criminality. While the first four categories were then collapsed into a more general category of multi-problematic relationships, the last two categories were collapsed under the category of antisociality. Table 1 describes the distribution of these categories in this study sample.

**Table 1:** Sample description

	Perpetrators^a^ (*n* = 77)	Victims (*n* = 86)
Age mean (*SD*)	46.87 years (*SD* = 17.11)	47.02 years (*SD* = 21.56)
Nationality		
Italian	76.8%	74.4%
Foreigner	23.2%	25.6%
Employment		
Employed	46.2%	56.4%
Unemployed	53.8%	43.6%
Profession		
Qualified	38.5%	20.5%
Unqualified	7.7%	35.9%
Criminal careers^b^		
Previous convictions	24.7%	
No previous convictions	75.3%	
Victim type^c^		
Known (*n* = 64)		76.2%
Unknown (*n* = 20)		23.8%
Type of link with victim %		
Familiar or acquaintance (*n* = 57)		67.9%
Stranger (*n* = 6)		7.1%
Prostitutes^d^ (*n* = 21) of whom:		25.0%
Italian		42.9%
Foreigner		57.1%
Relationship^e^		
Intimate relationship^f^ (*n* = 49) of whom:		58.3%
wife, daughter, and mother-in-law		- 2.04%
wife and daughters		- 2.04%
partner and friend		- 2.04%
Acquaintance (*n* = 29)		34.5%
Stranger (*n* = 6)		7.1%
Motives of femicide of whom^g^:		
passion killing (*n* = 25):		30.5%
against intimate victim		96.0%
against non-intimate victim		4.0%
family problems (*n* = 18):		22.0%
against intimate victim		88.9%
against non-intimate victim		11.1%
consequence of another crime (*n* = 14):		17.1%
against intimate victim		7.1%
against non-intimate victim		92.9%
predatory (*n* = 13):		15.9%
against intimate victim		–
against non-intimate victim		100%
loss of control (*n* = 6):		7.3%
against intimate victim		66.7%
against non-intimate victim		33.3%
mental disorder (*n* = 6):		7.3%
against intimate victim		66.7%
against non-intimate victim		33.3%

Percentages exclude missing values

^a^In three cases, the perpetrator had two victims; in three other cases, the perpetrator had three victims

^b^With the concept of “criminal careers” is meant here the official previous crimes and convictions attributed to the individual perpetrator, as indicated in the forensic files examined. We are aware that this is only a partial perspective of what a criminal career is. Albeit scientifically important, the study of criminal careers of femicide perpetrators was beyond the scope of this study. For further details on the criminal career paradigm, see the specialized literature [16, 64–66]

^c^According to the victimology literature, a victim is considered “known” if the perpetrator and the victim knew each other for at least 24 h prior to the femicide, while a victim is considered “unknown or stranger” if the victim did not know the offender (or vice versa) 24 h before the femicide. Some of the prostitute victims were killed by their habitual clients (known victims), but in other cases, it was difficult to establish if they knew each other for less than 24 h (unknown victims). In two instances, it was not possible to establish whether victims and perpetrators knew each other (missing data)

^d^The proportion of victims who practiced “prostitution” as a profession. In two cases (9.5%), the women were having an intimate relationship with their perpetrator and also were living together. In one case, the perpetrator felt emotionally attached to the victim, who did not reciprocate the interest

^e^This dimension involved three subcategories of relationship: affective and intimate; acquaintance or superficial; stranger or unknown

^f^In three cases (6.12%), IPF were characterized by multiple killings that followed soon after the murder of the partner/wife. In the case in which the killing involved the murder of only the mother, it was reported that the perpetrator had a complex and ambivalent relationship with her. They were living together

^g^Motives of femicide are comprised of six subcategories that find support in the typology literature on batterers [58, 67] and on domestic violence [17, 43] that helps to distinguish between IPF and NPF

These categories were developed according to the typologies available in the literature that distinguished different subtypes based on the severity and frequency of IPV, the generality of violence (intrafamilial or extrafamilial), and the perpetrator’s psychopathology or personality disorders [17, 67] and antisociality [12, 68, 69].

Data collected were coded on the basis of the presence (1) or absence (0) in the corresponding “motives of crime” category. When a discrepancy emerged, the two independent raters discussed the case with the research group and reassessed it, until a better level of agreement was reached. Cohen’s kappa statistic [70] provides a measure of agreement between raters that takes into account chance levels of agreement, and it is appropriate for this type of data. The agreement for the category “motives of crime” was highly significant (Cohen’s *K* was 0.92, *p* < 0.0001; 95% CI = − 0.036–0.101), as it was for the category “contentiousness” (Cohen’s *K* was 0.90, *p* < 0.0001; 95% CI = − 0.084–0.123) and for the category “overkilling” (Cohen’s *K* was 0.81, *p* < 0.0001; 95% CI = − 0.075–0.180) suggesting, according to Viera and Garrett [71] and to McHugh [72], a substantial interrater agreement coefficient for all of these variables.

### Sample

The sample involved in this study included 86 cases of femicide: in 89.5% of them (*n* = 77), the perpetrator killed a single victim, and in the remaining 10.5% of the cases (*n* = 9), he killed multiple victims. In 24.7% of the cases (*n* = 19), perpetrators had previous convictions. Data related to stalking were gathered for 56 cases; 17.9% (*n* = 10) of the perpetrators had been previously reported to the police for stalking.^5^

70.9% (*n* = 61) of the femicide cases examined were solved, with the conviction of the perpetrator, while 26.7% of the cases (*n* = 23) are still unsolved (cold cases). Because of new evidence, in 2.3% of the cases (*n* = 2), a new investigation was required by the General Prosecution Office.

The mean age of perpetrators was 46.87 years (*SD* = 17.11); perpetrators were mostly Italian (76.8%; *n =* 43) and unemployed (53.8%; *n* = 28) at the time of the femicide. When employed, they had mainly a qualified profession (e.g., manager, teacher, and civil servant) (38.5%; *n* = 20). The mean age of victims was 47.02 (*SD* = 21.56), so not significantly different from the age of their perpetrators (*t*(126.032) = 0.045, n.s.).

Victims were predominantly Italian (74.4%; *n* = 64), and in 43.6% of the cases (*n* = 34), they were unemployed, and when employed, they were involved in unskilled jobs (e.g., cleaning job and shop assistant) (*n =* 28; 35.9%), while in 20.5% of the cases (*n* = 16), victims were involved in a qualified profession (e.g. teacher and civil servant). Twenty-five percent of victims (*n* = 21) were prostitutes; in three cases, they were emotionally involved with their perpetrators, and in two cases, they were also living together. See Table 1 for details regarding the demographic characteristics of perpetrators and victims in this study.

### Analytical strategy

Descriptive analyses with chi-square and odds ratio (OR) were carried out to explore characteristics of the sample involved. The OR was calculated to identify which factors significantly and independently explained motives for killing and which other factors predicted the dynamics for killing up to extreme killing, i.e., overkilling. Also explored was whether contentiousness was a significant variable affecting the likelihood of IPF. The OR provides information about the existence, direction, and strength of an association between the target and comparison groups regarding the likelihood of an event occurring [73]. When ORs are higher than 1, situations characterized by that particular attribute have relatively higher odds of occurring than those that do not have that attribute.

## Results

### Temporal trends, time, and place of femicide

**Fig. 1:**
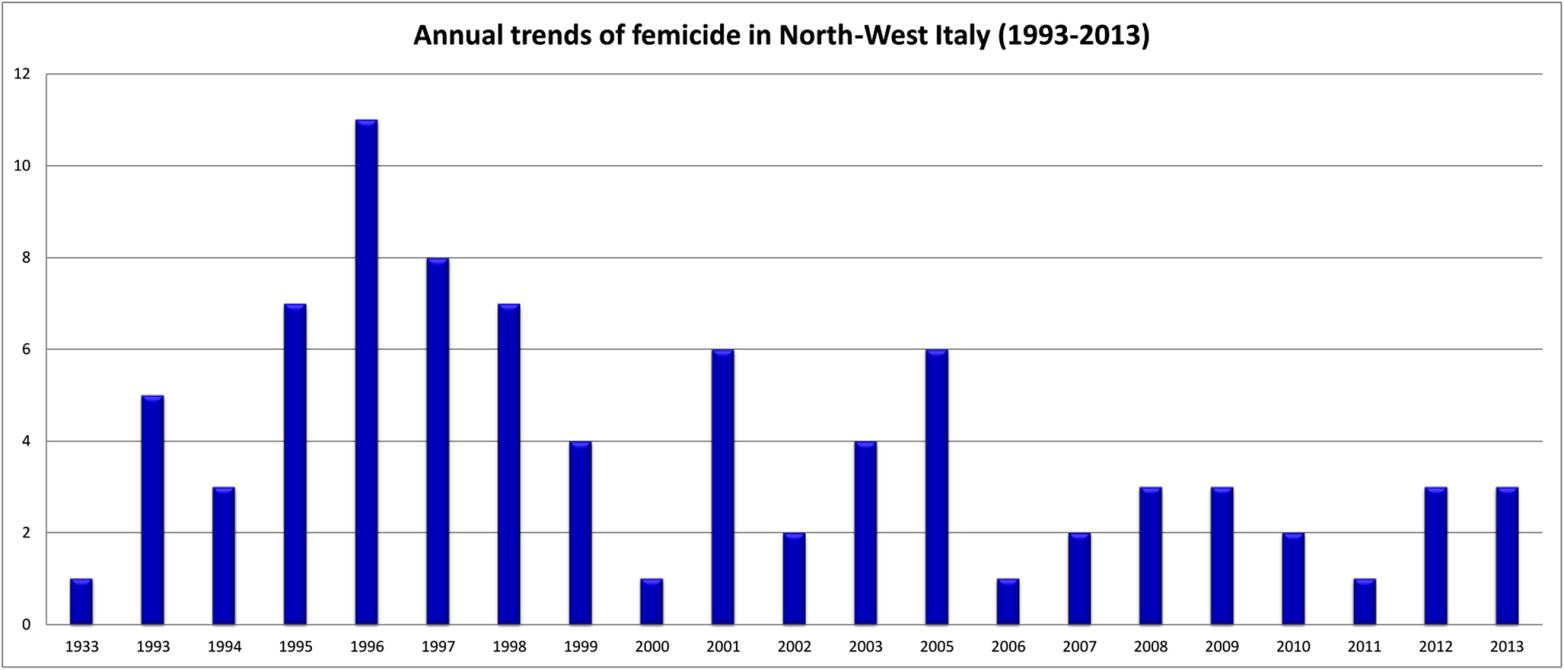
Annual trends of femicide in North-West Italy (1993–2013)

As shown in Fig. 1, femicides were not equally distributed within the temporal window (1993–2013) considered in this analysis; during 2004, no cases of femicide were recorded. 71.1% (*n* = 59) of the femicides occurred between 1993 and 2003: in particular, the highest concentration of femicides (42.2%; *n =* 35) took place in the least recent period, from 1993 to 1995 and 1996 to 2000 (24.1%; *n =* 20), while the lowest prevalence was recorded in 2001–2005 (15.7%; *n =* 13) and 2006–2013 (18.1%; *n =* 15). In three cases, it was impossible to establish the precise date of the killing. Furthermore, femicide in this study did not occur homogeneously during the day: it was observed that in 56.9% (*n* = 41) of the cases, women were likely to be killed during the day, between 5.00 am and 5.59 pm.

The majority of femicides took place at the victim’s or perpetrator’s house (69.4%; *n* = 59), while in 30.6% of the cases (*n* = 26), they occurred in a public place (such as car, street, or countryside). Findings suggest that, immediately after the murder, the perpetrator abandoned the crime scene (48.3%; *n* = 14), while in 34.5% of the cases (*n* = 10), he continued to interfere with rage against the victim’s body in the attempt to destroy any evidence of the murder. In 17.2% of the cases, the perpetrator denied any involvement with the crime (*n =* 5), and in 16.9% of the cases (*n* = 13), the perpetrator committed suicide after the femicide. Nearly all of these perpetrators (*n* = 11; 84.6%) committed suicide after having killed their current wives. In only one case (7.7%) was the victim the ex-wife, and in the other case, the victim was the mother with whom the perpetrator had a close and ambiguous relationship (see Table 1 for details).

### Medicolegal aspects and weapons

The most frequently hit body area was the head, which represented the location of the deadly injuries in 65.8% of cases (*n* = 52); in 12.7% of the cases (*n* = 10), the trunk was hit, and in 21.5% of the cases (*n* = 17), both body areas were targeted. The extent of damage and physical trauma caused by the application of mechanical force against the victim was so extremely severe that the victim endured complete disfiguration (64.3%; *n* = 54). Defense injuries were present in 61.9% of the victims (*n* = 26) as a natural reaction of an assault of any kind.

Findings suggest the presence of a prevalence of stabbing weapons or firearms in 51.9% of the cases (*n* = 42), while in 48.1% of the cases (*n* = 39), the perpetrator used a blunt object or his own hands to kill the victim. Furthermore, in the 13 cases in which the information was available, the firearms or stabbing weapons were formally registered^6^ in 53.8% of the cases (*n* = 7), while in 46.2% of the cases (*n* = 6), the perpetrator did not possess a regular license. Given the types of injuries inflicted, it was reasonable to establish that in 76.9% (*n* = 10) of the cases, the perpetrators seemed to have handled the weapon with particular dexterity to kill the victims.

It was also analyzed whether there was an association between the type of weapons used to kill and the type of relationship between victims and perpetrators (known and unknown). It was found that the likelihood of using a blunt object or bare hands was higher when perpetrators killed unknown victims (88.2%; *n* = 15) than when perpetrators killed known victims (38.1%; *n* = 24) (OR = 0.082; 95% CI = 0.017–0.391). In 61.9% of these cases (*n* = 39) when firearms or stabbing weapons were used, the perpetrators knew their victims.

Furthermore, the types of weapon differed depending on the level of intimacy between victims and perpetrators. When the relationship was intimate and emotionally intense, it was more likely that a stabbing weapon or a firearm was used to kill (66.7%; *n* = 32) in comparison with those cases in which the relationship was superficial (28.1%; *n* = 9) (OR = 0.196; 95% CI = 0.074–0.520).

### Relationships between victims and perpetrators: how and why were they linked?

As seen in Table 1, in 76.2% of the cases (*n* = 64), the victims knew their perpetrators, suggesting that femicide did not occur within an anonymous setting. To explore whether the types of relationship had some implication on femicide, data were analyzed according to whether the relationship was intimate or superficial: in 58.3% of the cases (*n* = 49), there was an intimate or a strong emotional attachment between victims and perpetrators, given that the victim was related to her perpetrator by marriage or by strong family ties. In some cases, the perpetrators killed multiple victims (i.e., daughters and mother-in-law) after having killed his wife first. In one instance, the perpetrator killed his mother: she was living with him, and in the forensic report, the relationship was described as emotionally ambiguous and intense. In 34.5% of the cases (*n* = 29), the relationship was characterized by a more superficial level of relationship (e.g., neighbors or acquaintances), and in 7.1% (*n* = 6) of the cases, the perpetrator was a complete stranger.

In those cases in which it was possible to establish how long victims and perpetrators knew each other (*n* = 51), the duration of the relationships lasted on average 16.22 years, reaching a peak of 62 years (*SD* = 15.83). However, the length of the relationship was not homogeneous; in fact, in 37.3% of the cases (*n* = 19), the relationship spanned between 1 day and 4.9 years; in another, 45.1% of the cases (*n* = 23) lasted over 20.0 years, and in the remaining 17.6% of the cases (*n* = 9), the relationship spanned between 5 and 19 years.

In 38.5% of cases (*n* = 25), victims and perpetrators lived together when the IPF took place; in 10.1% of the cases (*n* = 7), the IPF occurred after their cohabitation was interrupted, while in 50.1% of the cases (*n* = 33), they had never lived together.

### Motives behind IPF and NPF

**Fig. 2:**
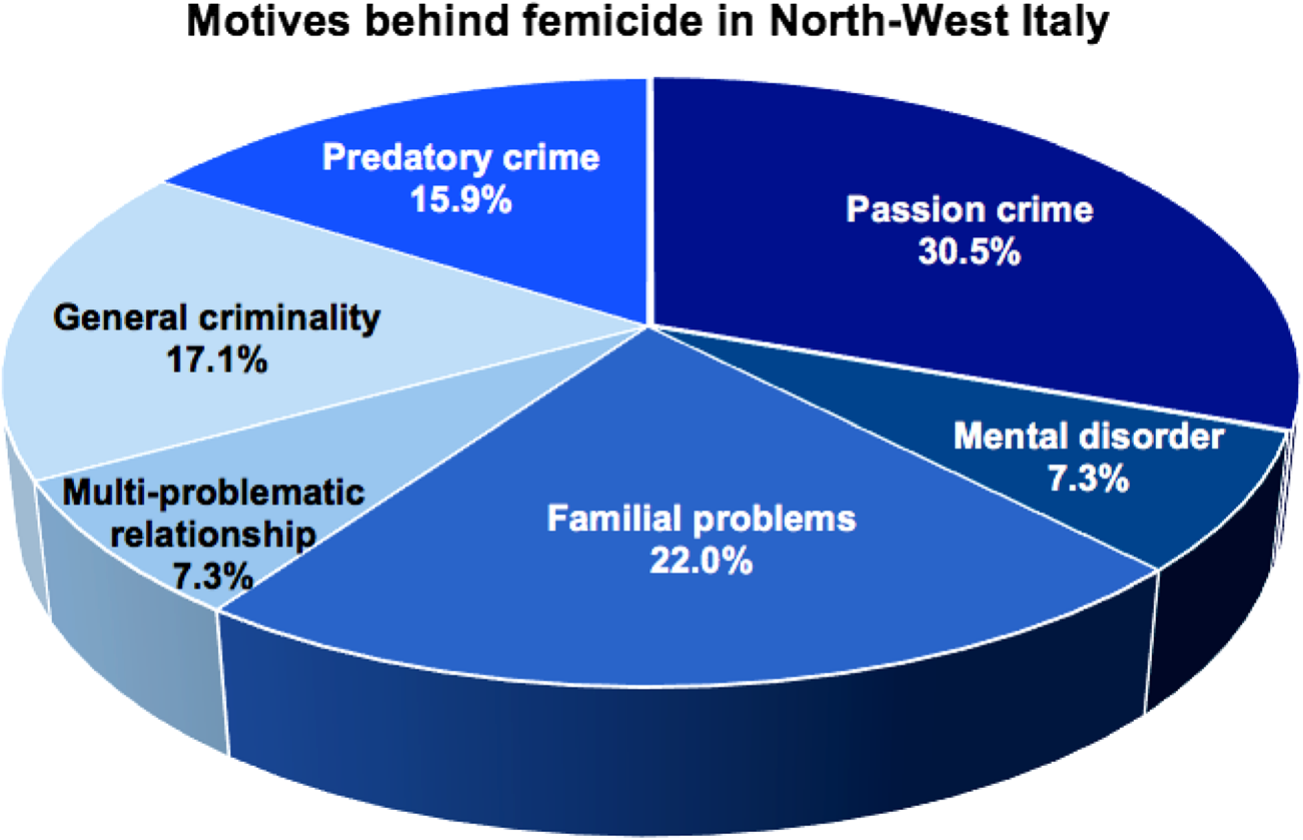
Motives behind femicide in North-West Italy

Regarding the motives behind femicide, data were analyzed according to the six categories identified, as shown in Fig. 2. The interest was to explore whether there was any difference in why intimate and non-intimate victims were more likely to be killed. Passion killing occurred in 30.5% of the cases (*n* = 25); IPF motivated by familial problems took place in 22.0% of the cases (*n* = 18). In 7.3% of the cases (*n* = 6), the victim was killed after a dramatic row with the perpetrator who then lost control and reacted with lethal rage. A predatory motive behind the NPF was identified in 15.9% of cases (*n* = 13), while in 17.1% of cases (*n* = 14), the victim was killed as a consequence of another crime committed. In a rather small percentage, 7.3% of femicide (*n* = 6) were associated with a mental disorder of the perpetrator. This last result is in line with what happened in North-East Italy [53] and with other clinical studies, which show that mental disorder could be a risk factor for interpersonal violence. However, most victims of IPF and NPF are not killed by men who suffered from a mental disorder [75] and who were not criminally responsible [76].

In order to analyze further the association between motives and types of relationships, two macro-categories seemed to synthesize best the findings. In 59.8% (*n* = 49) of cases, a multi-problematic relationship between victims and perpetrators, aggravated by health problems, economic difficulties, or continual contentiousness between partners, seemed to have lethally triggered the IPF, while in 40.2% of cases (*n* = 33), the NPF was driven by antisociality and general criminality (e.g., femicide as a consequence of another crime). Overall, it was found that 26.2% (*n* = 22) of femicides were sexual in nature.

According to these motives, the types of relationship between the victim and the perpetrator (affective versus superficial) seemed to have played a significant role in the IPF: the likelihood for a woman to be killed by a known person with whom there was (or had been) an affective relationship, albeit multi-problematic or perhaps because of it, was 50 times higher than the likelihood of being killed by an antisocial acquaintance (OR = 64.69; 95% CI = 14.81–282.51). 91.8% of the women (*n* = 45) were killed by men with whom they had a relationship, in comparison with 8.2% of those (*n* = 4) killed by an antisocial acquaintance.

This was the case when controlling for the emotional intensity and closeness of the relationship (intimacy versus distance): the likelihood of being killed by a person with whom the victim was intimately bonded was higher than being killed by an acquaintance (OR = .020; 95% CI = 0.005–0.077). 89.8% of the victims (*n* = 44) were, at the moment of the killing, enduring (or had endured in the past) a multi-problematic intimate relationship with their perpetrator in comparison with 10.2% (*n* = 5) of those who were, or had been, emotionally distant from him. On the contrary, 84.8% (*n* = 28) of those who were killed for antisocial or criminal motives were not involved in any kind of relationship with their perpetrator versus 15.2% (*n* = 5) of those who, albeit intimate with their perpetrator, were killed for criminal motives (e.g., robbery).

### Contentiousness

We explored this aspect further by looking at the extent to which the intimate relationship was marked by contentiousness between the victim and the perpetrator. According to the clinical files available, in 46.1% of the cases (*n* = 35), the relationship between victims and perpetrators was overshadowed by constant contentiousness. In such a relational climate, the likelihood of becoming a victim of IPF was significantly higher than in those cases in which the victims and the perpetrators were only superficially related (OR = 0.060; 95% CI = 0.016–0.229). The more superficial the relationship, the less likely there was contentiousness between partners that led to IPF.

### Overkilling

Overkilling describes the extent to which a victim is killed with ferocity and repeated damaging actions even after the cessation of vital life functions. In this sample, overkilling was present in 45.2% (*n* = 38) of femicide cases. Findings show that a considerable amount of multiple and severe wounds on the victim’s body were present. Overkilling was significantly correlated with the extension of damage and physical trauma caused to the victim’s body (rho = 0.628, *p* < 001).

Contrary to expectations and other studies [35], these findings suggest that differences in overkilling between intimate victims (52.1%; *n* = 25) and strangers or acquaintances (37.1%; *n* = 13) were only near statistical significance (OR = 1.84; 95% CI = 0.756–4.48).

### Risk of lethal violence against prostitutes

The majority of the prostitute victims were foreigners (57.1%; *n =* 12), and prostitution was their main professional occupation at the time of the femicide. Prostitutes were significantly younger (M = 33.62; *SD* = 15.68) than the other victims (M = 51.53; *SD* = 21.68) (*t*(44.081) = 4.029, *p* < 0001), suggesting a medium effect size [77] (*r* = 0.43). Moreover, prostitutes were more likely to be killed in secluded public areas (e.g., streets, city outskirts) (66.7%; *n* = 14) rather than in indoor locations (e.g., brothels, saunas, and parlors) in comparison with non-prostitute victims (19.0%; *n* = 12) (OR = 0.118; 95% CI = 0.039–0.355).

14.3% of the prostitutes (*n* = 3) were killed by a man with whom they had had an intimate relationship in comparison with 73.0% (*n* = 46) of non-prostitutes killed by their intimate partners (OR = 0.062; 95% CI = 0.016–.236). Antisocial motives (e.g., predatory killing) were, in fact, more likely to be those behind the murder of prostitutes (78.5%; *n* = 15) in comparison with non-prostitutes (28.6%; *n* = 18) (OR = 9.38; 95% CI = 2.74–32.11). The risk of being a victim of a sexually motivated crime was almost 14 times higher for prostitutes (66.7%; *n* = 14) in comparison with non-prostitutes (12.7%; *n* = 8) (OR = 13.75; 95% CI = 4.26–44.38).

## Discussion

The aim of this study was to explore femicide that occurred in North-West Italy between 1993 and 2013, in order to be able to identify risk factors that were at the basis of IPF and NPF and also to analyze types of relationships between victims and perpetrators and motives behind the crime.

Findings suggest that femicide was disproportionately perpetrated by intimate partners with whom the victims had a relationship. According to previous research carried out in North America [78–80], Europe [81–83], and Italy [35, 53, 55], femicide rarely occurred in an anonymous vacuum. In the majority of cases, it was in fact the epilogue of an abusive relationship with a high level of contentiousness between the victim and the perpetrator, which had become the independent and significant precursor that led IPV to escalate into IPF.

This is the first study in Italy that explores contentiousness and overkilling among IPF and NPF victims. Previous studies [35] show that the more intimate the relationships between the perpetrator and the victim, the higher the risk of overkilling. Albeit the result was only near statistical significance, over half of the IPF victims in this study were overkilled by a man with whom they were emotionally close. Victims were more likely killed in a familial setting, usually in their own home and with the use of firearms or stabbing weapons. According to the forensic pathology investigation, IPF victims seemed to have endured considerable physical trauma and damages associated with the violence, suggesting that it would have been very unlikely for them to have survived had medical attention been there earlier.

More studies are certainly necessary to examine further the extent to which the types of relationship, and the intensity of emotions involved, may play a significant role in how the victims is killed or overkilled. Research has in fact shown that women killed by an intimate partner scored higher in retrospectively measured risk for IPV than survivors [45]. This is why identifying predictors of IPV and IPF should become a priority in any government agenda so as to support further research in the field.

It is interesting to observe that this study shows the least concentration of femicide in North-West Italy in the most recent years (2005–2013) (28.9%; *n* = 24), in comparison with the earlier years (1993–2003) (71.1%; *n* = 59). A possible explanation could lie in the reinforced policy measures that may have become more effective in preventing the violence from escalating into femicide. It would also be possible that while women were less likely to be killed in North-West Italy, they were instead forced to endure a long-lasting and hidden pattern of abuse within the relationship. Eurostat statistics show a decrease in homicides in European countries over the past decades, while the rates of femicide tend to stay stable [84]. Contrary to the media that presents a quite alarming situation (e.g., the *availability heuristic*) [85], these data show a reduction in the number of femicides over time, which is coherent with other psycho-criminological studies [86, 87] and criminal data in Italy and in the Western world.

To give sense to these findings, the 86 femicides in North-West Italy were compared with 34 femicides examined in the district of Udine, North-East Italy, in the same temporal frame [53]. In North-East Italy, it was observed that 15 cases of femicides occurred between 2009 and 2013, and the rest of the cases were spread between 1994 and 2004 [53]. The small sample size and some differences in the data available made it quite difficult to attribute these differences to a possible transformative process of violence going on or to claim that some social aspects had a differential influence in these two Italian areas.

Both studies show that women in Italy were more likely to be killed by a man they knew and had an intimate relationship with. Intimate victims were more likely to be killed after an escalating row or from an attempt to end the relationship. In most cases for which the information was present, it emerged that femicide was a *by-product* of the relationship between victims and perpetrators. More studies are needed to explore the interactive combination of criminogenic and victimogenic factors in order to differentiate the level of IPF risk depending on the type and intensity of the relationship.

Though findings on femicide in this study confirmed those of North-East Italy for types of victims and perpetrators involved, types of weapons, and motives, some differences emerged due also to the data used. Variables such as contentiousness and overkilling were not explored in the study of Moreschi and colleagues [53]. It was also difficult to reach the conclusion as to whether IPV was more frequent in North-West than in North-East Italy. In order to make a comparative analysis, risk assessment programs should be organized to create uniformity in the way IPV and IPF are targeted within and between regional areas.

More empirical studies seem to be needed to test the assumption whether we are now facing a transformation in how intimate violence is occurring. *Is there a shift from a more direct and lethal force used, as the one shown in earlier periods in time, to more covert, indirect, and manipulative forms of control and violence?* If what is happening is that fewer women are killed, but more women are abused within the private space of intimate relationships, then different coordinated efforts from experts and institutions should take place to unveil the causes of IPV in all its forms and to assess the differential risk for IPV and IPF.

Contrary to the common belief that isolated places are more dangerous and that strangers mostly attack at night, the majority of IPF in North-West Italy took place during the day, in a domestic setting, and the majority of the victims had an intimate relationship with their killer. Findings also show that the kind of weapon used differed depending on the type of relationship between the victim and the perpetrator. The different weapons used to kill unknown victims (e.g., a blunt object) in comparison with known victims (e.g., firearms) may suggest various possible interpretations. The fact that unknown victims were more likely to be killed with bare hands or a blunt object might suggest that the killing was an unplanned and impulsive act. It might also suggest that the perpetrator was more likely to be vicious and aggressive, or to be large in size, so as to be able to carry out the blunt force murder. Firearms and stabbing weapons were used more frequently when the victim was known and when there was an intimate relationship with her. This finding might suggest that these weapons are more likely used when the killing is planned. Other studies have suggested that IPF are usually not spontaneous acts of rage or impulsive gestures but planned attacks [88].

Moreover, other research findings have shown that perpetrators who used guns inflicted the worst damage on their victims [89, 90] and acted out their violence after a long period of abuse and threat [91]. Regarding the locations of injuries on victims’ bodies, in this study, the head was the predominant target chosen by perpetrators to kill their victims, which may represent the easiest option to put a direct end to any reaction or to stop any defensive response from a person. In 61.9% of cases, victims presented defense injuries in an attempt to escape. This may also suggest that in only a small proportion of cases, victims were caught off guard by the attack and died without trying to defend themselves.

Motives of femicide were in line with those recognized in other studies [28, 51, 53]. Most cases of IPF were characterized by multi-problematic relationships, with jealousy, possessiveness, infidelity, and non-consensual separation between partners leading the man to kill. Only a marginal proportion of cases were triggered by mental illness; the association between mental illness and femicide was uncommon in this study, as in other scientific studies [75, 92], far from the widespread and generalized idea that IPF is a gesture of madness performed by the perpetrator. A sexual motive was present in over a quarter of the IPF cases examined, and the vast majority of these victims were prostitutes. It is difficult to draw any conclusion about this result, though it might be assumed, on the basis of other studies on violence against prostitutes [93, 94], that the killing might have represented the elimination of a sexual disposable commodity^7^ rather than a tentative one of simply eliminating a witness who had also been sexually abused.

According to previous research [95–97], which suggests that prostitutes who solicit on the streets are more at risk than those who work indoors, these results confirm this trend, making the street an extremely at-risk setting for these women, contrary to non-prostitute victims for whom “home” is, instead, the setting with the highest risk of IPF. Moreover, the antisocial and sexual motives behind the killing of prostitutes may suggest that some of the men who killed prostitutes are habitually violent and more likely to be antisocial [98].

These findings should be interpreted in light of a few limitations of the study.

All data were retrospective, and it was not possible to gather first-hand information from family members about the quality of the relationship between the victim and the perpetrator and from perpetrators about the motives behind the killing. The evidence gathered explains only part of the dynamics of the IPV that fostered the femicides.

Furthermore, it was impossible, with these data, to reconstruct exactly whether the violence was mostly unilateral (from man toward woman) and to identify the victimogenic factors that interacted with other factors to escalate into femicide.

Though femicide is often assessed within a gender perspective [99, 100], it was difficult in this study to identify those cases in which the motive of femicide was purely gender—when the woman is killed because she is a woman. This motive might have been the one that triggered those cases in which victims and perpetrators did not know each other, but it was impossible to reach this conclusion. In those cases in which the perpetrator was a total stranger, or when the victims were prostitutes, it cannot be excluded that the deadly violence had been motivated by misogyny.

## Conclusions

Femicide represents one of the most grievous challenges that our society faces. Femicide does not happen in an anonymous and unemotional setting [101], making it more likely to escalate up to IPF. Studies suggest [62, 81, 102] that violence against women is more likely to occur when a relationship between a man and a woman exists and when this relationship is intimate and emotionally close, rather than superficial, albeit there are cases in which the victim and her perpetrator are total strangers [16, 28]; the risk factors and processes implicated are likely to differ [12, 62, 82]. Behind the immediate damage (i.e., the death of the victim), it is likely that a continuous and escalating period of abuse, violence, psychological terrorism [43], contentiousness, and suffering had been building up to the climax of IPF [102]; in most cases, it is likely that the contentiousness in the relationship is kept secret by the victim in fear of retaliation.

If the question to ask experts is how to make intimate violence assessment more effective so as to prevent IPV from occurring in the short–medium term and to prevent it from escalating up to IPF in the long term, the onus that should be placed on governments and stake holders is to support scientific research. Research has recognized identifiable risk factors and processes for IPV and IPF [28] that should foster joint scientific, interdisciplinary, and interprofessional efforts to combat them efficiently and as early as possible.

## Footnotes

1 In this study, the general term femicide is used to imply the murder of a woman by any male perpetrator. The terms IPF and NPF are used when specifically referring to the murder of a woman by an intimate or a non-intimate partner perpetrator, respectively. In the “Materials and methods” and “Results” sections of this article, the differences in motives, dynamics, and weapons used to kill the victim are described and reported according to IPF and NPF, respectively.

2 Starting from the joint effort in Europe by the Council of Europe that led on 11 May 2011 to the *Convention on preventing and combating violence against women and domestic violence*, called also Istanbul Convention (2011), the European Union and the United Nations are now collaborating on a new global initiative called “Spotlight” [50], with the aim of eliminating all forms of violence against women: this project represents an effort to achieve gender equality in line with the 2030 Agenda for Sustainable Development and shows Europe’s constant commitment to this issue.

3 A legal enforcement process against women discrimination and protection of women’s rights was achieved, passing through different stages, from New Family Law called the “Reform on Family Law” (law n. 151/1975), Divorce Law called “Discipline of cases of dissolution of marriage” (law n. 898/1970), Abortion Law called “Rules for the social protection of motherhood and the voluntary interruption of pregnancy” (law n. 194/1978), to the Abolition of Crimes of Honor called the “Repeal of the criminal relevance of the cause of honor” (law n. 442/1981) that led, in 2009, to the enactment of the law known as “Measures against persecutor acts” (see art. 612bis of the criminal code.). The art. 612bis was introduced with the decree, n. 11, 23 February 2009, and converted in law n. 38/2009, called “Persecutory Acts” (the so-called *Anti-Stalking Law*). Other measures were designed to tackle the problem of violence against women with Law Decree n. 93, 14 August 2013, converted with modifications into the law n. 119, 15 October 2013, called “Urgent provisions on security and the fight against gender-based violence and on civil protection and provincial commissioners.”

4 A “typology” is a system of groupings, the members of which are identified by postulating specified attributes that are “mutually exclusive and collectively exhaustive […]. Typologies […] are less durable than classifications in that their descriptions are accepted only to the degree that they continue to provide solutions to problems” [61].

5 In Italy the *Anti-Stalking Law* was formally enacted in 2009. Before that time this type of crime was under the more general criminal code of *Persecutory Acts*. See note 3 in this article.

6 The purchase of firearms or weapons is strictly regulated in Italy. Applicants must obtain a specific authorization from the police, along with a gun license certificate, which is issued after medical and psychological examinations and after a favorable approval by the legal regulatory authority for the search on the criminal records of the applicant [74].

7 One of the perpetrators in this study confessed that he preferably looked for ugly, fat, old, and southerner prostitutes who resembled his stepmother, who he deeply hated. In his own words, he defined prostitutes as “[…] animals; they are only women, and I wanted to do some sort of clearance […].” In their work on prostitute homicide, Salfati and colleagues [94] addressed this issue and cited how the British serial killer Sutcliffe (1984) described why he had killed his victims: “the women I killed were filth-bastard prostitutes who were littering the streets. I was just clearing up the place a bit […].” Sutcliffe’s quote can be retrieved from http://members.aol.com/gjsayer/hutson/quotes.htm#victims
